# A Novel Construction of Efficient Substitution-Boxes Using Cubic Fractional Transformation

**DOI:** 10.3390/e21030245

**Published:** 2019-03-05

**Authors:** Amjad Hussain Zahid, Muhammad Junaid Arshad, Musheer Ahmad

**Affiliations:** 1 Department of Computer Science, University of Management and Technology, Lahore 54000, Pakistan; 2 Department of Computer Science, University of Engineering and Technology, Lahore 54000, Pakistan; 3 Department of Computer Engineering, Jamia Millia Islamia, New Delhi 110025, India

**Keywords:** substitution box, cubic fractional transformation, block ciphers, security

## Abstract

A symmetric block cipher employing a substitution–permutation duo is an effective technique for the provision of information security. For substitution, modern block ciphers use one or more substitution boxes (S-Boxes). Certain criteria and design principles are fulfilled and followed for the construction of a good S-Box. In this paper, an innovative technique to construct substitution-boxes using our cubic fractional transformation (CFT) is presented. The cryptographic strength of the proposed S-box is critically evaluated against the state of the art performance criteria of strong S-boxes, including bijection, nonlinearity, bit independence criterion, strict avalanche effect, and linear and differential approximation probabilities. The performance results of the proposed S-Box are compared with recently investigated S-Boxes to prove its cryptographic strength. The simulation and comparison analyses validate that the proposed S-Box construction method has adequate efficacy to generate efficient candidate S-Boxes for usage in block ciphers.

## 1. Introduction

Cryptography helps individuals and organizations to protect their data. For this purpose, different symmetric and asymmetric ciphers have been designed. Symmetric ciphers possess simplicity and efficiency, and consume fewer computational resources as compared to asymmetric ciphers. Symmetric ciphers have two major categories as stream and block ciphers [1]. A stream cipher encrypts the plaintext in bit-by-bit fashion. On the other hand, a block cipher encrypts a plaintext block of a fixed size consisting of many bits simultaneously. Mostly, block ciphers are used for internet communication whereas stream ciphers are used in situations where computational resources are limited or efficiency is not the main concern.

A block cipher is particularly useful to achieve data confidentiality, which is one of the cryptography goals. It is considered as one of the most widely used tools for the provision of data security [2]. The most modern and popular symmetric ciphers, like AES, DES, Blowfish, RC2, RC5, and IDEA, are all block ciphers. Block ciphers are easily implemented, more general, and cryptographically stronger as compared to the stream ciphers [3]. Most of the block ciphers use either the Feistel structure or the substitution and permutation operations. Block ciphers based on the Feistel structure divide the plaintext block into two or more parts and perform different operations on these parts. DES, Blowfish, Camellia, Kasumi, RC5, TEA, 3DES, RC6, etc. are some example block ciphers that use the Feistel structure. Another type of popular block cipher is termed the substitution-permutation network (SPN) [4]. These ciphers convert plaintext blocks using sub-keys and different numbers of rounds into respective ciphertext blocks. In each round, along with other operations, substitutions and permutations are performed on the input bits. A substitution operation substitutes a block of bits with another block of bits using a substitution box (S-Box). A permutation operation changes the positions of the bits or bytes in the given input block. AES, SHARK, PRESENT, SQUARE, etc. are among the most popular block ciphers that use substitution and permutation operations. Among these, AES cipher is most widely used in real life applications and many researchers have proposed improvements and enhancements of it.

An S-Box is a crucial part of modern-day block ciphers and is used to create a muddled ciphertext from the given plaintext. An S-Box is one of the fundamental techniques used to provide candid confusion. Confusion is the complex relationship that must be established between the plaintext and the ciphertext [5]. The strength of the cipher is directly proportional to the level of confusion produced in the ciphertext. As a result, the cryptographic strength of a block cipher using an S-Box is dependent on the cryptographic strength of that S-Box. Many scholars have investigated and explored ways to propose quality S-Boxes and analyzed their strength against some standard criteria and benchmarks, such as nonlinearity (NL), bijection, strict avalanche criterion (SAC), bit independence criterion (BIC), linear probability (LP), and differential probability (DP) etc.

In [6], the authors proposed a mechanism to create repositories or a database of S-Boxes, which possess robust and resilient cryptographic topographies. These repositories can be of a great help to provide the security of data and information during the customization of the block ciphers. Mohamed et al. [7] proposed 18 S-Box properties to be present to resist against different types of cryptanalytic attacks. The presence of a greater number of properties in an S-Box makes it more secure. SAC, nonlinearity, BIC, and resistance to linear cryptanalysis and differential cryptanalysis are a few of the most needed S-Box criteria [8,9].

Generally, a block cipher consists of many parts. An S-Box, being the lone non-linear part of a block cipher, is very useful for enhancing the security of the plaintext by creating confusion in the ciphertext. The non-linearity provided by an S-Box offers defense against linear cryptanalysis [10]. Block ciphers use two types of S-Boxes: Static and dynamic. Ciphers employing static S-Boxes use the same S-Box in each round. This type of S-Box permits the attackers to investigate S-Box properties, find its weaknesses, and ultimately obtain the opportunity for cryptanalysis of the ciphertext [7,11,12]. For example, DES uses static S-Boxes. To overcome the problems of static S-Boxes and provide more security against cryptanalysis, many researchers have investigated new and novel ways to design S-Boxes, which are random, dynamic, and key-dependent. Dynamic S-Box generation based on the key ensures improvements in the strength of the cipher. For example, Blowfish cipher uses dynamic S-Boxes.

AES is one of the popular block ciphers which use S-Boxes in the encryption and decryption processes. Sahmoud et al. [13] proposed an enhancement to the security offered by AES. It uses multiple sub-keys for the encryption of different plaintext blocks. However, the new cipher is more complex and slow as compared to AES. Moh’d et al. [14] introduced AES-512, which is an enhancement to the original AES-128. It uses a plaintext block and a key of the size of 512-bits each. It has an increase of more than 200% in the throughput compared to the original AES-128. In [15], the authors proposed key based dynamic S-Boxes and increased the number of steps performed in each round of AES. The authors in [16,17] rectified and improved the AES cipher by optimizing the S-boxes. These optimized S-Boxes are more efficient than the traditional AES S-Box. Niemiec et al. [18] introduced a symmetric block cipher that uses key-dependent generic S-boxes. The authors described the way to generate a huge number of S-Boxes, which all exhibit good security landscapes. Kazlauskas et al. [19] proposed four algorithms to generate key-dependent S-Boxes and evaluated the worth of these boxes using distance metrics. Results demonstrate that the generated S-Boxes are of a high quality.

The Feistel structure has been used as the main construct in many of the symmetric ciphers, like DES, GOST, RC5, etc. DES and GOST each uses eight S-Boxes with sizes 6 × 4 and 4 × 4, respectively. The authors in [10] used the Feistel structure to choose a dynamic S-Box depending upon the plaintext/ciphertext. One of the 16 S-Boxes of the 4 × 4 size was chosen using 8-bits plaintext/ciphertext. The right 4-bits choose the S-Box and the left 4-bits act as the input to the S-Box. The authors also suggested a variation by dividing the 8-bits of the plaintext/ciphertext in two non-equal size parts and accordingly the size of the S-box and number of S-Boxes could be determined. This design of dynamic S-Box achieves the non-linear confusion and hence the cryptanalysis of the ciphertext becomes very difficult. Similarly, LiCi is a lightweight block cipher that uses the Feistel structure and active S-Boxes and is suitable for both software and hardware platforms, especially when energy is the main constraint [20]. It shows good resistance against many cryptanalytic attacks.

One of the desirable properties of modern block ciphers is the avalanche effect [21]. This property requires that a 1-bit change in key or the plaintext should produce substantial changes in the ciphertext. If the value of the avalanche effect is very small (at least 50% of the ciphertext bits are not changed), the block cipher is weak and cryptanalysis of the ciphertext becomes easy. The proposed cipher in [22] using a dynamic S-Box shows a good avalanche effect compared to the standard AES when a single bit is changed in the plaintext or the key. Shi et al. [23] analyzed in detail the avalanche effect of the AES S-Box and concluded that both S-Boxes of AES have a good avalanche effect. The authors in [21] analyzed five symmetric ciphers and concluded that AES has the highest avalanche effect. Mahmoud et al. [24] proposed a modification to generate an S-Box to be used for AES having a 128-bits key. The authors compared the correlation factor, avalanche effect, and time efficiency of the standard AES and the proposed one. The value of the correlation factor was between −0.3 and 0.3, which shows that the standard AES and proposed cipher have no dependence. The avalanche effect of the S-Box proposed in [24] has values between 0.41 and 0.61, which makes it resistant against linear and differential cryptanalysis. However, the standard AES is more efficient. Mar et al. [25] proposed three methods to evaluate and analyze the SAC of a given S-Box. These methods are very simple and check whether the given S-Box has an avalanche effect, possesses completeness, and is strong enough against cryptanalysis. Adams et al. [26] described an efficient process to generate S-Boxes, which possess the cryptographic properties of bijection, SAC, BIC, NL, etc. The authors also proved that a small change in the input guarantees nonlinearity and the inverse S-Boxes also fulfill the evaluation criteria. The generated S-Boxes are prospective contenders for SPN block ciphers.

Chaotic cryptography is among the most interesting areas in the field of information security in the recent era as the chaotic systems possess the property of randomness [27]. Many researchers have used chaos to design ciphers with good cryptographic strength. Garg et al. [28] analyzed different techniques to design an S-Box and concluded that S-Boxes designed using a chaotic approach demonstrate good cryptographic strength. The authors in [29] proposed some chaotic map-based block ciphers using a different approach and then showed that the proposed ciphers were resilient to different attacks. Ahmad et al. [30] designed an efficient S-Box using the chaos and travelling salesman problem and analyzed its performance against cryptographic standards. The results indicate that the proposed S-Box is more effective when compared to its counterparts. The authors in [31,32,33,34,35] proposed strong S-Boxes based on the combination of chaotic maps and other different algorithms. The evaluation of the cryptographic performance of these S-Boxes against NL, SAC, BIC, etc. revealed that hyperchaotic systems are stronger than chaotic ones. Peng et al. [36] and Solami et al. [37] devised methods to generate an S-Box using a hyperchaotic system. The resultant S-Boxes demonstrate a good response to cryptographic properties, like BIC, SAC, DP, etc. The suggested methods have the capability to generate a huge number of S-Boxes.

Another popular area in cryptography is DNA computing, which is being considered as a possible solution to the design of resilient ciphers. Kadhim et al. [38] and Al-Wattar et al. [39] proposed efficient S-Boxes using DNA computing, analyzed the security of the proposed ciphers using different criteria, and showed that the ciphers passed the test criteria. Many other researchers have used DNA computing to design and propose block ciphers, such as [40,41,42,43].

Ciphers using S-Boxes highly depend on the security of the S-Boxes. Thus, the identification of a tool to evaluate and find an S-Box with high security that can also assist in the design of efficient S-Boxes is considered critical. Wang et al. [44] developed a software tool to analyze and test the performance of S-boxes. The software tool supports the design of good block ciphers. Albermany et al. [45] suggested random block cipher (RBC). It uses a master key of a length of 128 bits and 64 bits plaintext. Eight sub-keys are generated from the master key. The size of each sub-key is 16 bits. A bijective function, a new S-box, and binary operations are employed in this cipher. Thirty-two S-boxes are used and the length of the ciphertext is 128 bits. It is able to encrypt a large amount of plaintext data very efficiently. Many other authors have contributed to the design of various S-Boxes using different techniques. Tran et al. [46] proposed an S-Box using graph isomorphism and showed that their S-Box exhibits the desired cryptographic properties. Coset diagrams are a special type of graph that has real life applications. Razzaq et al. [47] used the coset diagram to propose a new S-Box, evaluated its strength against cryptanalysis, and showed that the results were promising. Novel techniques of cryptanalysis reveal that there are still some scarcities in the existing ciphers [48]. To provide better security, the complexity of a cipher is increased and as a result the efficiency of the respective cipher decreases. The authors proposed an efficient and more secure block cipher, which uses operations, like linear and non-linear mixing, by employing S-Box, capsulation, etc. for confusion and diffusion effects.

Linear fractional transformation (LFT) is another area which helps in the generation of better S-Boxes. Farwa et al. [49] proposed a modest and proficient algorithm to generate an S-Box based on LFT. The authors analyzed the strength of their S-Box against cryptographic properties, like SAC, BIC, NL, LP, DP, etc. The authors in [50,51,52] proposed efficient algorithms to design good S-Boxes based on LFT and the projective general linear group on Galois field, respectively. The proposed S-Boxes showed good performances when compared with other existing S-Boxes. Many researchers have exposed the cellular automata field to propose S-Boxes [53,54]. Authors have shown that their S-Boxes have good strength when critically analyzed.

The techniques and methods for the generation of S-Boxes presented in the literature are either suitable for the creation of static S-boxes or are very complicated and time-consuming. Static S-Boxes have their own limitations and weaknesses. These S-Boxes may help attackers in the cryptanalysis of the captured ciphertext and hence they may reach the original plaintext. On the other hand, the methods presented in the literature that generate dynamic and key-dependent S-boxes are very complex and less efficient. Thus, the need for a simple and efficient method to generate dynamic S-Boxes exists.

In this paper, a novel design method for the construction of efficient S-Boxes for block ciphers is proposed. The following considerations were kept in mind while designing the proposed S-Box:

- An S-box that helps in the security enhancement of the block cipher and resists cryptanalysis;
- An S-Box that is simple to construct;
- An S-Box that is generated dynamically using sub-keys;
- An S-Box that fulfills the most needed S-Box criteria, like NL, SAC, BIC, LP, DP, etc.

The method proposed in this paper for the construction of an S-Box is an innovative one and is quite different from the approaches presented in the literature. A cubic fractional transformation is proposed for the construction of strong S-Boxes. After the S-Box was designed, a performance analysis was performed to show its strength. The proposed S-Box demonstrated a very good cryptographic strength when compared with other recently designed S-Boxes. The results indicated that the proposed S-Box is a good choice for block ciphers.

The structure of the rest of the paper is as follows. Section 2 of this paper presents the design architecture of the proposed S-Box. The performance evaluation of the proposed S-Box against its cryptographic properties is discussed in Section 3 and a comparison is made with some S-boxes. Section 4 concludes the research paper.

## 2. Proposed Substitution Box

Modern block ciphers employ byte substitution to replace a complete byte (one element) of a matrix with another complete byte using the substitution box (S-Box). Generally, the design of an S-Box involves nonlinear mapping, which results in bijection. Many researchers have designed S-Boxes that are cryptographically strong using such mappings. One such mapping is linear fractional transformation (LFT), which was exhaustively explored for the construction of the S-boxes [49,50,51,52]. However, the process of generating these S-Boxes using LFT is very complicated and time consuming.

**Figure 1:**
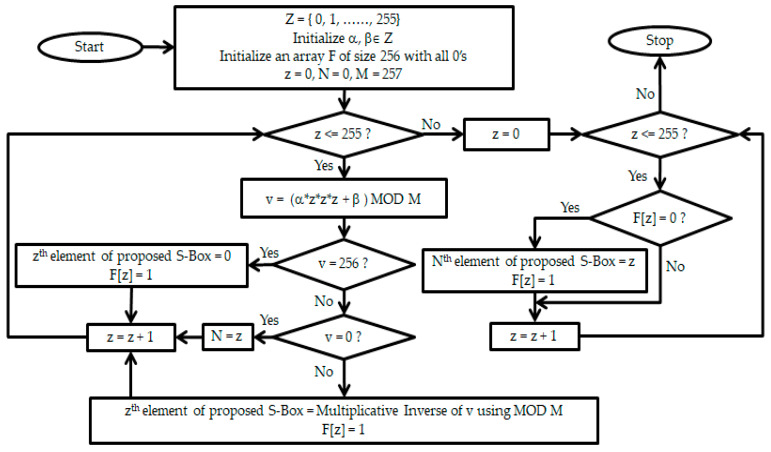
Flowchart for the construction of the proposed S-Box.

In this paper, we extend the idea of LFT and construct our new transformation to generate an S-Box using another nonlinear mapping method in a simple and efficient way. We call this extended transformation cubic fractional transformation (CFT). A cubic fractional transformation is a function of the form:

(1) $$C\left(z\right)=\frac{1}{\alpha {\left(z\right)}^{3}+\beta }\;\left(\mathrm{MOD}\left(2^{n}+1\right)\right)\;\;\alpha ,\beta ,z\in \;Z$$

where, *Z* = {0, 1, ……, 2*^n^* − 1}, both *α* and *β* are not 0 at the same time, and *α*(*z*)^3^ + *β* ≠ 0 is used to construct the *n* × *n* S-box. The nonlinear nature of CFT stimulates its usage in byte substitution. The procedure to generate the proposed S-Box for *n* = 8 is illustrated in Figure 1.

To elaborate the construction of the proposed S-Box using Equation (1), let us have a specific type of cubic fractional transformation as given in Equation (2). Let *Z* = {0, 1, …, 2*^n^* − 1} = {0, 1, …, 2^8^ − 1} = {0, 1, 2, …, 254, 255} for *n* = 8. Any values can be chosen for *α* and *β* (*α*, *β* ∈ *Z*) that gratify the condition of *α*(*z*)^3^ + *β* ≠ 0. For the sake of the calculations here, we have chosen *α* = 95 and *β* = 15. The CFT function, *C*(*z*), given in Equation (2) generates values of *Z* – {0, 106} when *z* ∈ *Z* – {176, 184}. When *z* = 176, *C*(*z*) evaluates to 256 ∉ *Z*. When *z* = 184, the denominator of Equation (2) evaluates to 0. To keep the function, *C*(*z*), bijective, we explicitly define *C*(*z*) for *z* ∈ {176, 184} as conditioned in Equation (2). An example S-Box of a size of 8 × 8 is generated using a CFT function, *c*: Z→Z, given as:

(2) C(z)={(195(z)3+15)(MOD 257)  if z∈Z−{176,184}   0  if z=176   106  if z=184

This particular cubic fractional transformation of Equation (2) generates the elements of our proposed S-Box, which are organized in a 16 × 16 matrix as shown in Table 1.

As mentioned above, any values for *α* and *β* (*α*, *β* ∈ *Z*) can be used in Equation (1) to generate an S-Box. One can choose sub-keys as the values for *α* and *β* to generate dynamic and key-dependent S-boxes.

## 3. Cryptographic Properties of the Proposed S-Box

In this section, we analyze our method and S-Box given in Table 1 against widely accepted standard S-Box performance criteria to gauge its cryptographic strength.

### 3.1. Bijection

A function, *f*: X→Y, is bijective if and only if ∀*_y_*_∈*Y*_, ∃ a unique *x* ∈ *X*, such that *f*(*x*) = *y*. For *n*-bit inputs, this property maps all possible 2*^n^* input values to distinct output values. In other words, when *x*_1_ ≠ *x*_2_, then *f*(*x*_1_) ≠ *f*(*x*_2_). All component Boolean functions (*f*_1_ to *f*_8_) of the proposed S-box are balanced (number of 1’s = number of 0’s). Further, all 2^8^ output values of the S-box are distinctive where each output value ∈ *Z* = {0, 1, …., 255}.

### 3.2. Nonlinearity

An S-Box operation should not be a linear mapping of an input to an output as it weakens the strength of any cipher. A high value of non-linearity provides resistance against linear cryptanalysis. The nonlinearity of an *n*-bit Boolean function, f, is calculated as [55,56]:

(3) $$NL\left(f\right)=2^{n-1}-\frac{1}{2}\left(\;\underset{z\in {\left\{0,1\right\}}^{n}}{\max}\left|W_{f}\left(z\right)\right|\right)$$

where, *W_f_*(*z*) = Walsh spectrum of the coordinate Boolean function, *f*, which is measured as:

$$W_{f}\left(z\right)=\underset{t\in {\left\{0,1\right\}}^{n}}{\sum }{\left(-1\right)}^{f\left(t\right)⊕t.z}$$

Here, *t.z* is the dot product of *t* and *z* in bit-by-bit fashion and *z* ∈ {0, 1}*^n^*. The nonlinearity values, *NL*(*f*), of the Boolean functions of our S-Box are given in Table 2.

**Figure 2:**
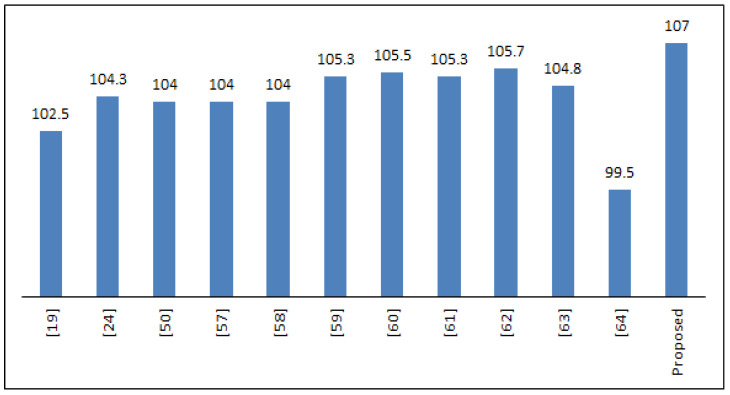
Average nonlinearity values of the proposed S-Box and other S-Boxes.

Table 3 and Figure 2 give a comparison between the nonlinearity values of the proposed S-box and various other S-Boxes. It is obvious from Table 3 and Figure 2 that our S-Box has a greater capability for diluting the linearity, making the linear cryptanalysis very challenging.

### 3.3. Strict Avalanche Criterion (SAC)

Webster et al. [65] introduced strict avalanche criteria as the important property for strong S-boxes. This property states that a single bit change in the input should change half of the output bits. An SAC value nearer to 0.5 is considered adequate. An S-Box should exhibit a strict avalanche effect to have good randomness. Table 4 provides the SAC values of our S-Box and it is obvious that the average SAC value of the proposed S-Box is equal to 0.5. This result indicates that our S-Box satisfies the SAC property very well. 

### 3.4. Bit Independence Criterion (BIC)

The authors in [65] introduced this criterion. According to this criterion, if an input bit, *x*, is inverted; this changes the output bits, *y* and *z*, independently. For greater security, efforts are made to decrease the dependence between output bits. If a given S-Box satisfies the BIC, all the component Boolean functions possess high nonlinearity and meet the SAC [65]. Table 5 and Table 6 demonstrate the possible values of nonlinearity and the SAC for the component Boolean functions of the proposed S-Box.

Considering the nonlinearities and SAC, the average BIC values are 103.5 and 0.5, respectively. If a given S-Box is non-linear and demonstrates the SAC, it fulfills BIC [26]. These values are an indication of a very week linear relationship between the output bits and hence fully justify the BIC of the proposed S-box.

### 3.5. Linear Probability

Modern block ciphers are designed to create as much diffusion and confusion of the bits as possible for the security of data and provide a shield against different approaches that cryptanalysts adopt to obtain the plaintext. Mostly, this is achieved by S-Boxes, which provide nonlinear transformations. If an S-Box is designed with a low linear probability (LP), it is a very good cryptographic tool against linear cryptanalysis.

The linear probability of an S-Box is calculated using the following equation [56]:

(4) $$LP=\underset{A_{x},\;B_{x}\neq 0\;}{\max}\left|\frac{\#\{x\in Z|x\cdot A_{x}=S\left(x\right)\cdot B_{x}\}}{2^{n}}-\frac{1}{2}\right|$$

where, *A_x_* and *B_x_* represent the input and output masks, respectively and *Z* = {0, 1, …., 255}. 

The maximum value of LP of our S-box is only 0.156, and thus our S-Box provides good resistance against linear cryptanalysis.

### 3.6. Differential Uniformity

Differential cryptanalysis is one of the most commonly used methods to reach the plaintext. Here, the differences in the original message (plaintext) and the differences in the ciphertext are obtained. The pairing of these differences may help reach some of the key values. To defy differential cryptanalysis, a small value of differential uniformity (DU) for a given S-Box is required. Differential uniformity is calculated as [56]:

(5) $$DU=\underset{\;A_{x}\neq 0,\;B_{x}}{\max}\left[\#\{x\in Z|S\left(x\right)⊕S\left(x\;⊕\;∆x\right)\;=\;∆y]\right\}\;$$

where, Δ*x* and Δ*y* are the input and output differentials, respectively. An S-box with smaller differentials is better at repelling differential cryptanalysis. Table 7 shows the differential uniformity values of the proposed S-box. The proposed S-box has a maximum value of DU as 10 and its count is only 7. So, the differential probability (DP) is 0.039. These smaller values of DU and DP provide evidence that the proposed S-Box has good resistance against differential cryptanalysis.

**Figure 3:**
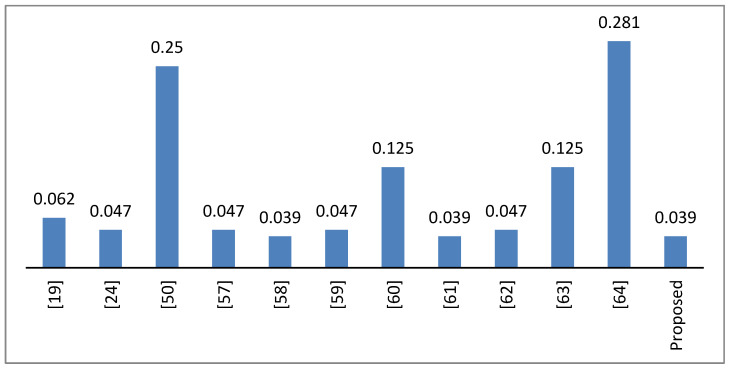
Differential probability values of the proposed and other S-Boxes.

Figure 3 shows a graphical comparison of the DP values of the proposed and other S-Boxes.

### 3.7. Performance Comparison

Table 8 shows the performance comparison of our S-Box with other S-Boxes based on the cryptographic properties. Our findings are:

- A high value of non-linearity provides resistance against linear cryptanalysis [55]. The average nonlinearity of the proposed S-Box is superior to the rest of the S-Boxes in Table 8. This results in decent confusion and makes the proposed S-box resilient against linear cryptanalysis.
- An SAC value near 0.5 (the perfect value for SAC) is the ultimate goal of every S-Box designer. Table 8 demonstrates that our SAC value (0.497) is very close to this perfect value. We can say that our S-Box satisfies the SAC.
- Similarly, the BIC value of our S-box is better than the BIC values of more than half of the other S-boxes. Table 5 and Table 6 demonstrate that the BIC values of our S-Box with respect to nonlinearity and SAC are adequate, thus satisfying the BIC test.
- Any S-Box with a lesser value of differential probability is more resilient against differential cryptanalysis. The DP value of our S-Box is 0.039, which is better than the DP values of nine other S-Boxes and equal to the DP values of two other S-Boxes as shown in Table 8. This value of DP reflects the strength of our S-Box.
- To defy linear cryptanalysis, a smaller value of LP for a given S-Box is desired by S-Box designers. The LP value of our S-Box is 0.156. Due to this small value, we can say that our S-box is resistant to linear cryptanalysis.

From the above comparison it is evident that our S-Box fulfills the most needed S-Box criteria and benchmarks, like SAC, BIC, NL, LP, DP, etc., and hence possesses better cryptographic strength.

## 4. Conclusions

In this paper, we proposed a new transformation and suggested a novel method to construct efficient S-Boxes using cubic fractional transformation. The security strength of the proposed S-Box was studied using different standard criteria. The simulation results were in accordance with other relevant S-Boxes, rationalizing the performance of our S-Box method. The performance of our S-Box was good in most of the cases when compared with other recent S-Boxes. In particular, the scores of the SAC, BIC, nonlinearity, LP, and DP of the proposed S-Box provide evidence for it as a new alternative in the S-Box design domain. The promising results of the proposed S-Box analysis make it a potential candidate for usage in modern-day block ciphers. It is worth mentioning that our method is the first to explore the cubic fractional transformation for S-Box construction. Stronger S-boxes using cubic fractional transformation, like the proposed S-Box, are expected to emerge for usage in practical systems for secure communication.

## Figures and Tables

**Table 1:** Proposed S-Box.

120	250	193	180	88	223	185	112	210	242	233	241	91	95	53	174
132	115	125	220	74	135	190	80	72	104	43	8	239	38	194	186
183	153	31	160	116	157	114	165	48	13	52	221	244	63	24	119
46	171	169	158	9	177	42	123	140	122	111	216	245	98	70	197
203	235	168	187	12	26	137	138	101	60	225	100	113	28	195	146
29	199	189	86	214	102	200	39	178	191	227	44	27	15	246	141
144	134	255	19	22	204	18	139	82	35	156	57	209	181	79	93
188	231	206	97	77	128	143	155	167	59	208	175	253	3	73	218
62	61	47	159	78	68	136	126	58	36	152	252	249	45	67	229
54	56	99	6	94	198	145	226	173	247	34	11	85	87	248	118
192	213	133	212	237	21	92	20	215	121	219	49	109	50	238	64
0	176	66	1	76	254	150	222	106	129	205	40	196	127	230	179
154	69	30	163	33	10	4	55	2	105	7	117	71	65	81	251
148	170	182	217	232	236	151	124	224	17	131	41	166	161	96	184
107	83	162	37	130	172	228	75	25	103	240	147	108	207	211	234
32	110	51	23	16	201	202	164	14	84	149	243	142	5	90	89

**Table 2:** Coordinate Boolean functions of the proposed S-box and their nonlinearity values.

*f*	*f*_1_	*f*_2_	*f*_3_	*f*_4_	*f*_5_	*f*_6_	*f*_7_	*f*_8_
*NL(f)*	106	106	106	108	108	108	108	106

**Table 3:** Comparison of the nonlinearity values of different S-boxes.

S-Box method	Minimum	Maximum	Average
[19]	98	108	102.5
[24]	96	110	104.3
[50]	98	108	104
[57]	98	108	104
[58]	102	106	104
[59]	102	108	105.3
[60]	100	110	105.5
[61]	104	106	105.3
[62]	100	108	105.7
[63]	100	108	104.8
[64]	94	104	99.5
Proposed	106	108	107

**Table 4:** SAC (strict avalanche criterion) values of the proposed S-box.

0.484	0.468	0.453	0.515	0.546	0.468	0.468	0.484
0.484	0.515	0.578	0.468	0.453	0.468	0.468	0.515
0.437	0.531	0.531	0.515	0.500	0.578	0.468	0.453
0.531	0.531	0.578	0.531	0.531	0.515	0.468	0.453
0.546	0.578	0.484	0.531	0.468	0.531	0.468	0.484
0.484	0.484	0.468	0.421	0.453	0.515	0.437	0.531
0.531	0.421	0.437	0.468	0.453	0.562	0.531	0.531
0.453	0.515	0.531	0.515	0.453	0.468	0.515	0.515

**Table 5:** BIC (bit independence criterion) and nonlinearity.

	*f*_1_	*f*_2_	*f*_3_	*f*_4_	*f*_5_	*f*_6_	*f*_7_	*f*_8_
*f*_1_	-	102	108	108	102	100	108	104
*f*_2_	102	-	104	102	108	108	104	100
*f*_3_	108	104	-	104	106	102	100	102
*f*_4_	108	102	104	-	98	104	98	102
*f*_5_	102	108	106	98	-	102	106	104
*f*_6_	100	108	102	104	102	-	104	106
*f*_7_	108	104	100	98	106	104	-	102
*f*_8_	104	100	102	102	104	106	102	-

**Table 6:** BIC and SAC.

	*f*_1_	*f*_2_	*f*_3_	*f*_4_	*f*_5_	*f*_6_	*f*_7_	*f*_8_
*f*_1_	-	0.521	0.521	0.519	0.507	0.501	0.484	0.523
*f*_2_	0.521	-	0.490	0.500	0.501	0.503	0.505	0.472
*f*_3_	0.521	0.490	-	0.509	0.505	0.517	0.472	0.500
*f*_4_	0.519	0.500	0.509	-	0.500	0.511	0.480	0.500
*f*_5_	0.507	0.501	0.505	0.500	-	0.517	0.507	0.509
*f*_6_	0.501	0.503	0.517	0.511	0.517	-	0.513	0.496
*f*_7_	0.484	0.505	0.472	0.480	0.507	0.513	-	0.513
*f*_8_	0.523	0.472	0.500	0.500	0.509	0.496	0.513	-

**Table 7:** Differential uniformity values of the proposed S-Box.

8	6	8	6	6	6	6	6	8	8	6	8	6	6	6	6
8	6	6	6	6	6	8	8	8	6	8	6	6	6	8	6
8	6	10	6	6	6	6	8	6	6	8	6	6	6	8	6
8	8	6	6	6	6	6	6	8	6	6	6	6	6	6	6
6	6	6	8	4	6	6	6	6	8	8	8	6	8	6	6
6	8	8	6	6	6	8	6	8	6	8	6	6	6	6	8
8	8	6	8	6	4	6	8	8	6	6	8	8	8	6	8
8	6	8	8	6	8	8	6	6	6	6	6	8	8	8	6
6	6	6	8	6	8	6	6	8	8	6	6	8	8	8	6
6	8	8	6	6	6	6	6	6	6	6	8	6	6	8	8
6	6	6	8	6	6	6	8	8	6	6	6	10	6	6	6
6	8	4	8	6	6	6	6	6	6	8	6	8	6	6	8
6	6	6	8	6	6	8	8	10	6	6	6	6	6	6	6
6	6	8	6	6	6	6	6	8	6	6	8	6	6	6	6
8	6	8	8	6	8	10	10	6	6	6	6	8	6	6	6
6	6	8	6	6	10	6	10	8	8	8	8	6	8	8	-

**Table 8:** Recital comparison of different S-Boxes. NL: nonlinearity; LP: linear probability; DP: differential probability.

S-Box Method	Average Nonlinearity	SAC	BIC-NL	LP	DP
[19]	102.5	0.492	103.3	0.141	0.062
[24]	104.3	0.497	103.4	0.133	0.047
[50]	104	0.505	103.4	0.133	0.250
[57]	104	0.507	102.9	0.086	0.047
[58]	104	0.498	102.9	0.148	0.039
[59]	105.3	0.502	103.7	0.125	0.047
[60]	105.5	0.499	106	0.133	0.125
[61]	105.3	0.504	104.6	0.133	0.039
[62]	105.7	0.498	104.3	0.109	0.047
[63]	104.8	0.501	105.1	0.125	0.125
[64]	99.5	0.516	101.7	0.132	0.281
Proposed	107	0.497	103.5	0.156	0.039

## References

[1] Bhanot R., Hans R. (2015). A Review and Comparative Analysis of Various Encryption Algorithms. Int. J. Secur. Its Appl..

[2] Paar C., Pelzl J., Preneel B. (2010). Understanding Cryptography.

[3] Shamir A. Stream Ciphers: Dead or Alive? In Proceedings of the 10th International Conference on Theory and Application of Cryptology and Information Security, Jeju Island, Korea, 5–9 December 2004.

[4] Lambić D., Živković M. (2013). Comparison of Random S-Box Generation Methods. De L’institut Mathématique.

[5] Lauridsen M.M., Rechberger C., Knudsen L.R. (2016). Design and Analysis of Symmetric Primitive.

[6] Dragomir I.R., Lazăr M. Generating and Testing the Components of a Block Cipher. Proceedings of the 18th International Conference on Electronics, Computers and Artificial Intelligence.

[7] Mohamed K., Nazran M., Pauzi M., Hani F., Ali H.M., Ariffin S., Huda N., Zulkipli N. Study of S-box Properties in Block Cipher. Proceedings of the International Conference on Computer Communication and Control Technology.

[8] Manjula G., Mohan H.S. Constructing Key Dependent Dynamic S-Box for AES Block Cipher System. Proceedings of the International Conference on Applied and Theoretical Computing and Communication Technology.

[9] Radhakrishnan S.V., Subramanian S. An Analytical Approach to S-box Generation. Proceedings of the International Conference on Communication and Signal Processing.

[10] Du Z., Xu Q., Zhang J., Li M. Design and Analysis of Dynamic S-Box based on Feistel. Proceedings of the International Conference on Advanced Information Technology, Electronic and Automation Control.

[11] Katiyar S., Jeyanthi N. (2016). Pure Dynamic S-box Construction. Int. J. Comput..

[12] Alabaichi A., Salih A.I. Enhance Security of Advance Encryption Standard Algorithm Based on Key-dependent S-Box. Proceedings of the International Conference on Digital Information Processing and Communications.

[13] Sahmoud S., Elmasry W., Abudalfa S. (2013). Enhancement the Security of AES against Modern Attacks by Using Variable Key Block Cipher. Int. Arab J. e-Technol..

[14] Moh’d A., Jararweh Y., Tawalbeh L. AES-512: 512-Bit Advanced Encryption Standard Algorithm Design and Evaluation. Proceedings of the International Conference on Information Assurance and Security.

[15] Juremi J., Mahmod R., Sulaiman S. A Proposal for Improving AES S-box with Rotation and Key-Dependent. Proceedings of the International Conference on Digital Cyber Security, Cyber Warfare and Digital Forensic.

[16] Sahoo O.B., Kole D.K., Rahaman H. An optimized S-box for Advanced Encryption Standard (AES) design. Proceedings of the International Conference on Advanced Computer Communication.

[17] Wang H., Zheng H., Hu B., Tang H. Improved lightweight encryption algorithm based on optimized S-box. Proceedings of the International Conference on Computational and Information Sciences.

[18] Niemiec M., Machowski Ł. A new symmetric block cipher based on key-dependent S-boxes. Proceedings of the International Conference on ultra-Modern Telecommunications and Control Systems.

[19] Kazlauskas K., Smaliukas R., Vaicekauskas G. (2016). A Novel Method to Design S-Boxes Based on Key-Dependent Permutation Schemes and its Quality Analysis. Int. J. Adv. Comput. Sci. Appl..

[20] Patil J., Bansod G., Kant K.S. LiCi: A new ultra-lightweight block cipher. Proceedings of the International Conference on Emerging Trends and Innovation in ICT.

[21] Agrawal H., Sharma M. (2010). Implementation and analysis of various symmetric cryptosystems. Indian J. Sci. Technol..

[22] Nejad F.H., Sabah S., Jam A.J. Analysis of Avalanche Effect on Advance Encryption Standard by using Dynamic S-Box Depends on Rounds Keys. Proceedings of the International Conference on Computational Science and Technology.

[23] Shi H., Deng Y., Guan Y. Analysis of the Avalanche Effect of the AES S Box. Proceedings of the International Conference on Artificial Intelligence, Management Science and Electronic Commerce.

[24] Mahmoud E.M., Hafez A.A., Elgarf T.A., Zekry A.H. (2013). Dynamic AES-128 with Key-Dependent S-box. Int. J. Eng. Res. Appl..

[25] Mar P.P., Latt K.M. (2008). New Analysis Methods on Strict Avalanche Criterion of S-Boxes. Int. J. Math. Comput. Sci..

[26] Adams C., Tavares S. (1990). The Structured Design of Cryptographically Good S-Boxes. J. Cryptol..

[27] Ou C.M. (2008). Design of Block Ciphers by Simple Chaotic Functions. Comput. Intell. Mag..

[28] Garg S., Upadhyay D. (2013). S-Box Design Approaches: Critical Analysis and Future Directions. Int. J. Adv. Res. Comput. Sci. Electron. Eng..

[29] Jakimoski G., Kocarev L. (2001). Chaos and Cryptography: Block Encryption Ciphers Based on Chaotic Maps. IEEE Trans. Circuits Syst. I Fundam. Theory Appl..

[30] Ahmad M., Mittal N., Garg P., Khan M.M. (2016). Efficient Cryptographic Substitution Box Design Using Travelling Salesman Problem and Chaos. Perspect. Sci..

[31] Ahmad M., Haleem H., Khan P.M. A New Chaotic Substitution Box Design for Block Ciphers. Proceedings of the International Conference on Signal Processing and Integrated Networks.

[32] Ahmed H.A., Zolkipli M.F., Ahmad M. (2018). A novel efficient substitution-box design based on firefly algorithm and discrete chaotic map. Neural Comput. Appl..

[33] Ahmad M., Doja M.N., Beg M.M.S. (2018). ABC Optimization Based Construction of Strong Substitution-Boxes. Wirel. Pers. Commun..

[34] Alzaidi A.A., Ahmad M., Doja M.N., Solami E.A., Beg M.M.S. (2018). A New 1D Chaotic Map and beta-Hill Climbing for Generating Substitution-Boxes. IEEE Access.

[35] Alzaidi A.A., Ahmad M., Ahmed H.S., Solami E.A. (2018). Sine-Cosine Optimization-Based Bijective Substitution-Boxes Construction Using Enhanced Dynamics of Chaotic Map. Complexity.

[36] Peng J., Jin S., Lei L., Jia R. (2016). A Novel Method for Designing Dynamical Key-Dependent S-Boxes based on Hyperchaotic System. Int. J. Adv. Comput. Technol..

[37] Solami E.A., Ahmad M., Volos C., Doja M., Beg M. (2018). A New Hyperchaotic System-Based Design for Efficient Bijective Substitution-Boxes. Entropy.

[38] Kadhim A., Majeed G.H.A. Proposal New S-Box Depending on DNA computing and Mathematical Operations. Proceedings of the International Conference on Multidisciplinary in IT and Communication Science and Applications.

[39] Al-Wattar A.H., Mahmod R., Zukarnain Z.A., Udzir N.I. (2015). A New DNA-Based S-Box. Int. J. Eng. Technol..

[40] Leier A., Richter C., Banzhaf W., Rauhe H. (2000). Cryptography with DNA Binary Strands. BioSystems.

[41] Rahman N.H.U., Balamurugan C., Mariappan R. (2016). A Novel DNA Computing based Encryption and Decryption Algorithm. Procedia Comput. Sci..

[42] Raj B.B., Vijay J.F., Mahalakshmi T. (2016). Secure Data Transfer through DNA Cryptography using Symmetric Algorithm. Int. J. Comput. Appl..

[43] Shaw H. (2017). A Cryptographic System Based upon the Principles of Gene Expression. Cryptography.

[44] Wang Y., Xie Q., Wu Y., Du B. A Software for S-box Performance Analysis and Test. Proceedings of the International Conference on Electronic Commerce and Business Intelligence.

[45] Albermany S.A.K., Hamade F.R., Safdar G.A. New Random Block Cipher Algorithm. Proceedings of the International Conference on Current Research in Computer Science and Information Technology.

[46] Tran B.N., Nguyen T.D., Tran T.D. A New S-Box Structure Based on Graph Isomorphism. Proceedings of the International Conference on Computational Intelligence and Security.

[47] Razaq A., Yousaf A., Shuaib U., Siddiqui N., Ullah A., Waheed A. (2017). A Novel Construction of Substitution Box involving Coset Diagram and a Bijective Map. Secur. Comm. Netw..

[48] Al-Hazaimeh O.M.A. (2013). Design of a New Block Cipher Algorithm. Netw. Complex Syst..

[49] Farwa S., Shah T., Idrees L. (2016). A Highly Nonlinear S-Box based on a Fractional Linear Transformation. SpringerPlus.

[50] Hussain I., Shah T., Gondal M.A., Khan M., Khan W.A. (2011). Construction of New S-box using a Linear Fractional Transformation. World Appl. Sci. J..

[51] Altaleb A., Saeed M.S., Hussain I., Aslam M. (2017). An Algorithm for the Construction of Substitution Box for Block Ciphers based on Projective General Linear Group. AIP Adv..

[52] Sarfraz M., Hussain I., Ali F. (2016). Construction of S-Box Based on Mobius Transformation and Increasing its Confusion Creating Ability through Invertible Function. Int. J. Comput. Sci. Inf. Secur..

[53] Gangadari B.R., Ahamed S.R. (2016). Design of cryptographically secure AES like S-Box using second-order reversible cellular automata for wireless body area network applications. Healthc. Technol. Lett..

[54] Picek S., Mariot L., Yang B., Jakobovic D., Mentens N. Design of S-boxes defined with Cellular Automata Rules. Proceedings of the ACM International Conference on Computing Frontiers.

[55] Cusick T.W., Stanica P. (2009). Cryptographic Boolean Functions and Applications.

[56] Biham E., Shamir A., Menezes A.J., Vanstone S.A. Differential Cryptanalysis of DES-like Cryptosystems. Advances in Cryptology-CRYPT0’ 90.

[57] Alkhaldi A.H., Hussain I., Gondal M.A. (2015). A novel design for the construction of safe S-boxes based on TDERC sequence. Alex. Eng. J..

[58] Chen G. (2008). A novel heuristic method for obtaining S-boxes. Chaos Solitons Fractals.

[59] Belazi A., Rhouma R., Belghith S. A novel approach to construct S-box based on Rossler system. Proceedings of the International Wireless Communications and Mobile Computing Conference.

[60] Mahmood S., Farwa S., Rafiq M., Riaz S.M.J., Shah T., Jamal S.S. (2018). To Study the Effect of the Generating Polynomial on the Quality of Nonlinear Components in Block Ciphers. Secur. Commun. Netw..

[61] Siddiqui N., Afsar U., Shah T., Qureshi A. (2016). A Novel Construction of S16 AES S-boxes. Int. J. Comput. Sci. Inf. Secur..

[62] Hussain I., Shah T., Gondal M.A., Wang Y. (2011). Analyses of SKIPJACK S-Box. World Appl. Sci. J..

[63] Hussain I., Shah T., Gondal M.A., Khan W.A., Mahmood H. (2013). A group theoretic approach to construct cryptographically strong substitution boxes. Neural Comput. Appl..

[64] Hussain I., Shah T., Gondal M.A., Mahmood H. (2011). Some analysis of S-box based on residue of prime number. Proc. Pak. Acad. Sci..

[65] Webster A.F., Tavares S.E. On the Design of S-Boxes. Proceedings of the Conference on Theory and Application of Cryptographic Techniques.

